# Targeting MK2 Is a Novel Approach to Interfere in Multiple Myeloma

**DOI:** 10.3389/fonc.2019.00722

**Published:** 2019-08-08

**Authors:** Mengjie Guo, Dongdong Sun, Zhimin Fan, Yuxia Yuan, Miaomiao Shao, Jianhao Hou, Yuqi Zhu, Rongfang Wei, Yan Zhu, Jinjun Qian, Fei Li, Ye Yang, Chunyan Gu

**Affiliations:** ^1^The Third Affiliated Hospital of Nanjing University of Chinese Medicine, Nanjing, China; ^2^School of Medicine and Life Sciences, Nanjing University of Chinese Medicine, Nanjing, China; ^3^The First Clinical Medical College, Nanjing University of Chinese Medicine, Nanjing, China; ^4^Department of Hematology, The First Affiliated Hospital of Nanchang University, Nanchang, China; ^5^School of Holistic Integrative Medicine, Nanjing University of Chinese Medicine, Nanjing, China

**Keywords:** multiple myeloma, MK2, inhibitor, proliferation, combination

## Abstract

MAPKAPK2 (MK2), the direct substrate of p38 MAPK, has been well-acknowledged as an attractive drug target for cancer therapy. However, few studies have assessed the functions of it in multiple myeloma (MM). In the present study, MK2 expression of MM patients was analyzed by gene expression profiling (GEP) and array-based comparative genomic hybridization (aCGH). Several experiments *in vitro* including MTT assay, Western blot and flow cytometry analysis were performed to identify the function of MK2 in MM. In addition, we conducted mouse survival experiments to explain the effects of MK2 on MM *in vivo*. mRNA level of MK2 and chromosomal gain of MK2 locus in MM cells significantly increased compared to normal samples. Furthermore, MM patients with high expression of MK2 were associated with a poor outcome. Follow-up studies showed that MK2 exerted a remarkably positive effect on MM cell proliferation and drug-resistance. Further exploration focusing on MK2 inhibitor IV revealed its inhibitory action on MM growth and drug-resistance, as well as improving survival in mouse models. In addition, a combination of MK2 inhibitor IV and the key MM therapeutic agents including bortezomib, doxorubicin, or dexamethasone facilitated curative effects on inhibiting MM cell proliferation. Taken together, our study reveals the clinical relevance of MK2 inhibition in MM and demonstrates that targeting MK2 may afford a new therapeutic approach to MM.

## Introduction

Multiple myeloma (MM) is the second most prevalent hematologic malignancy derived from neoplastic growth of bone marrow plasma cells. Chemotherapy has been reported to interfere with the function of cellular machineries (like DNA replication and cell division), and is regarded as one of the useful therapeutic methods for MM; thus more and more novel agents are developed and tested (1). The National Comprehensive Cancer Network (NCCN) delineated the treatment guideline that lenalidomide and bortezomib regimens involving combination therapy with doxorubicin and/or dexamethasone are currently recommended in front-line treatment paradigm for MM (2). Although the survival outcomes of MM patients have been improved by new therapeutic approaches, it is still far away from having satisfactory effects on chemotherapy and relapse remains the major obstacle in MM management, which makes MM incurable (3, 4).

MAPK-activated protein kinase 2 (MK2), a serine/threonine-protein kinase known as the best-understood downstream partner of p38 MAP kinase, is involved in many cellular processes such as stress and inflammatory responses, nuclear export, cell proliferation and invasion (5). A meta-analysis demonstrated that there is a relatively higher expression of MK2 in MM compared with normal plasma cells by the means of ONCOMINE microarray database (6). In particular, overexpression of MK2 could be linked with MM drug resistance and relapse, due to the fact that p38-MAPKAPK2-Hsp27 signaling preserves the survival of cancer stem cells (6). Consequently, it is conceivable that MK2 may act as a powerful hallmark of cancer cells and inhibiting MK2 could disrupt tumor growth (7), which provides new avenues to explore the response of cancers to their therapeutic agents.

As reported, MK2 inhibitors exhibited desirable efficacy without toxic side effects, indicating that they are promising candidates for improving cancer treatment clinically. To this end, the research focused on this issue has been developed (8). MK2 inhibitor IV is a non-ATP competitive MK2 inhibitor and an excellent pharmacologic tool for specifically testing MK2 biology, because of its high degree of selectivity (9). However, little attention has been paid to MK2 inhibitor IV on MM. In addition, as inactivation of MK2 could enhance bortezomib cytotoxicity (10), it is an interesting investigation to explore whether the combination of MK2 inhibitor IV and current chemotherapeutics could achieve synergistic anti-tumoral effects on MM. Several studies have assessed the role of MK2 in chemotherapy, however seemingly conflicting results occur (11, 12). Thus, it is of great importance to choose the suitable chemotherapeutic drug combined with MK2 inhibitors for cancer treatment.

In the present study, we characterized MK2 expression in MM cells compared to normal control cells to reveal the relationship among MK2 expression, clinical characteristics and overall survival in total therapy 2 (TT2). Then the functions of MK2 in MM cell proliferation and drug-resistance were explored. Furthermore, we provided *in vitro* data declaring the anti-proliferation efficacy of MK2 inhibitor IV in MM cells and gained further insights to determine whether MK2 inhibitor is more effective than monotherapeutic agent (such as bortezomib, doxorubicin, or dexamethasone). Additionally, combination of MK2 inhibitor IV and bortezomib, doxorubicin or dexamethasone presented better inhibition effect on MM cell proliferation.

## Materials and Methods

### Cell Lines and Cell Culture

Human MM cell lines, APR1, and OCI-MY5 cells were maintained in RPMI 1640 (Gibco, Grand Island, NY) and supplemented with 10% fetal bovine serum (FCS) (Gibco, Grand Island, NY), penicillin and streptomycin solution (100 μg/mL, Sigma, St. Louis, MO) under the condition of 37°C in a humidified atmosphere of 5% CO_2_.

### Reagents

MK2 antibody, MK2 Clustered Regularly Interspaced Short Palindromic Repeats (CRISPR) lentiviral activation particles, and MK2-shRNA lentiviral particle were purchased from Santa Cruz Biotechnology (Dallas, Texas). Caspase-3, PARP, β-actin, and pAKT antibodies were obtained from Cell Signaling Technology (Danvers, MA). Trypan Blue, dexamethasone and doxorubicin were from Sigma (St. Louis, MO). Bortezomib was purchased from Selleck Chemicals (Houston, TX). MK2 inhibitor IV was purchased from Cayman Chemical (Ann Arbor, MI).

### Cell Growth Assays

Following cell transfection or 48 h after different drug treatment, MK2 inhibitor IV IC_50_ 15 μM; bortezomib IC_50_ 80 nM; dexamethasone IC_50_ 25 μM; doxorubicin IC_50_ 1.5 μM; MK2 inhibitor IV IC_25_ 4.75 μM + bortezomib IC_25_ 46.2 nM; MK2 inhibitor IV IC_25_ 4.75 μM + dexamethasone IC_25_ 6.6 μM; MK2 inhibitor IV IC_25_ 4.75 μM + doxorubicin IC_25_ 0.3 μM. Cell growth was evaluated using MTT assay according to the method described in the literature (13). In short, cells were seeded at 4,000~5,000 cells/well in 96-well plates, 10 μL of MTT and 90 μL of complete medium were added to each well according to the manufacturer's instructions, and the culture plate was incubated at 37°C for 4 h. Then, the culture medium was removed and 150 μL dimethyl sulfoxide was added to each well. After vigorous shaking for 10 min, the absorbance was read by a fluorometer (CytoFluor; Applied Biosystems, Foster City, CA, USA) at 570 nm.

### Cell Proliferation

Cells were enumerated using a hemocytometer. The fraction of dead cells was determined by trypan blue staining.

### Flow Cytometry

A flow cytometry assay was applied to analyze the cell cycle distribution as previously reported (14). Briefly, samples were washed with PBS and stained with PI solution (Yeasen, Shanghai, China) for 30 min. All samples were analyzed using FlowSight (Merck Millipore, Darmstadt, Germany).

### Western Blot Analysis

Western blots were utilized to measure the protein levels of MK2, pAKT and apoptotic markers in MM cells. In brief, around 20 μg protein per sample was extracted and whole-cell lysate were electrophorezed by 4–12% SDS polyacrylamide gel electrophoresis (SDS-PAGE) and transferred to nitrocellulose. After incubation with primary and second antibodies, blots were subsequently stripped and re-probed for β-actin as protein controls. The immunostaining band was quantified using Image J software.

### Soft Agar Clonogenic Assay

Clonogenic formation was monitored by plating 10,000 MM cells in 0.5 mL 0.33% agar RPMI 1640 medium (Invitrogen, Carlsbad, CA) with 10% FBS in 12-well plate. The cells were incubated at 37°C with 5% CO_2_ and fed by the same medium for 1–2 week, twice per week. The colonies were imaged and colony numbers were counted by Image J.

### Apoptosis and Dye-Efflux Multidrug-Resistance Assays

Drug-affected apoptosis following exposure to bortezomib or doxorubicin was detected in MM cells using flow-cytometric Annexin V apoptosis detection kit APC (catalog number 88–8007) from eBioscience (San Diego, CA). In brief, one million cells were washed and suspended in 1 mL binding buffer, followed by adding the Annexin V-APC (5 μL) to the cells suspension (100 μL). Then, cells were incubated for 15 min at room temperature, washed and re-suspended in binding buffer. The labeled cells were analyzed via flow cytometry.

The eFluxx-ID™ Multidrug resistance assay was applied to measure drug resistance as previously described (15). Shortly, 500,000 cells were incubated for 30 min under the circumstance of 37°C in water bath with detection reagent, Golden dye. Afterwards, cells were washed and re-suspended in ice-cold PBS for flow analysis, and MCF7 cells were used as reference.

### 5TMM Mouse Model

5TMM3VT murine myeloma cells (1 × 10^6^) were injected subcutaneously into the abdomen of 6 weeks old C57BL/KaLwrij mice (Jackson laboratory, Bar Harbor, Maine) (*n* = 20). After 2 days, MK2 inhibitor IV treatment used on 10 mice and intraperitoneally injected at the dose of 20 mg/kg three times a week lasting for 78 days until all the mice were dead (Tuesday, Thursday, and Saturday). The time of death caused by paralysis in the control group and the MK2i group was recorded in turn, and the survival curve was drawn at the end (16). All experimental procedures were in compliance with the National research council guidance's for the care and use of laboratory animals and approved by Animal Ethical and Experimental Committee of Nanjing University of Chinese Medicine (ACU170501).

### Statistical Considerations

All data was shown as means ± SD. Two experimental groups were analyzed by Student's *t*-test, while multiple (*n* ≥ 3) groups were analyzed with one-way ANOVA. The correlation of MK2 with disease progression, event-free and overall survivals were measured using the Kaplan-Meier method. Significance was set at *p* < 0.05.

## Results

### Amplification of MK2 Is Relevant for Poor Survival in MM Patients

**Figure 1:**
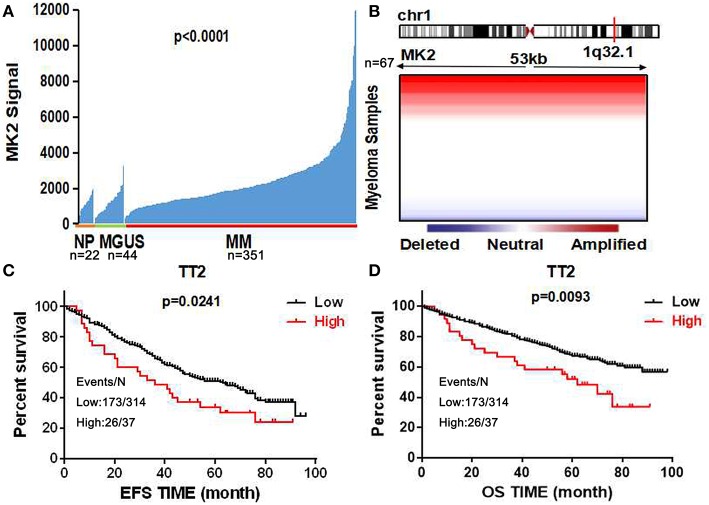
MK2 acts as a poor prognostic marker in MM. **(A)** MK2 expression of normal plasma cells (NP, *n* = 22), monoclonal gammopathy of undetermined significance cells (MGUS, *n* = 44), and myeloma patient plasma cells (*n* = 351) in GEP dataset. **(B)** Heatmap illustrating MK2 copy number variation in 67 primary MM samples. **(C,D)** Kaplan-Meier analysis on the event free survival **(C)** and overall survival **(D)** of MM patients according to the MK2 expression in TT2. Data are expressed as mean ± SD.

Using the gene expression profiling (GEP) database collected from NIH Gene Expression Omnibus GSE2658 as a discovery tool, we found that MK2 expression levels exhibited a dramatic upward trend from normal plasma cells (NP, *n* = 22), monoclonal gammopathy of undetermined significance cells (MGUS, *n* = 44), to newly diagnosed myeloma patient plasma cells (*n* = 351) (Figure 1A). The analysis of array-based comparative genomic hybridization (aCGH) data showed that MK2 locus was amplified in MM patient samples to a major extent (Figure 1B). To correlate with clinical parameters (Table 1), MK2 expression was impressively associated with clinical parameters like CRP at least 4.0 mg/l (*p* < 0.05), chromosomal abnormalities (by G-banding) (*p* < 0.05), and MRI focal bone lesions (*p* < 0.05). Furthermore, Kaplan-Meier survival curves showed that increment of MK2 expression was linked with an apparently shorter response duration of both event free (EF) and overall survival (OS) in TT2 (Total Therapy 2) cohort, respectively (Figures 1C,D). Continuing our previous study focusing on the relationship between MK2 and outcomes of MM patients in the APEX cohort, which was an independent cohort of MM patients (17), we further confirmed that MK2 acts as a valuable diagnostic and prognostic marker in MM.

**Table 1:** The Correlation of MK2 Expression and Clinical Characteristics in TT2.

**Characteristics**	**High MK2**	**Low MK2**	***p*-value**
	**(%, *n* = 32)**	**(%, *n* = 319)**	
Age at least 65 years	10.8	23.2	NS
Female sex	43.2	43.3	NS
White race	81.1	89.5	NS
IgA isotype	18.9	26.4	NS
CRP at least 4.0 mg/l	13.5	5.1	<0.05
β2-Microglobulin at least 4.0 mg/l	48.6	32.8	0.055
Hemoglobin <10 g/dl	27	24.8	NS
Albumin <3.5 g/dl	35.1	36.6	NS
Creatinine at least 2.0 mg/dl	16.2	10.5	NS
Chromosomal abnormalities (by G-banding)	54.1	33.1	<0.05
MRI focal bone lesions, at least three	73	54.5	<0.05
LDH at least 190 IU/l	32.4	34.1	NS
Hyperdiploid	29.7	17.2	NS
Hypodiploid	21.6	14.6	NS
Amplification of 1q21	54.1	44.9	NS

### Alteration in MK2 Expression Is Crucial for MM Cell Growth and Drug-Resistance

**Figure 2:**
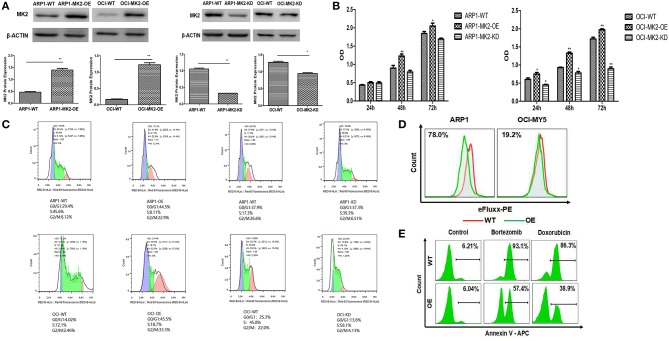
MK2 overexpression promotes proliferation and drug-resistance in MM. **(A)** Validation of MK2 expression levels in MK2-overexpression (OE) and MK2-knockdown (KD) MM cells compared with untransfected cells (WT). **(B)** MTT assay on MK2-overexpression (OE) and MK2-knockdown (KD) ARP1 and OCI-MY5 cells compared with untransfected cells (WT). **(C)** The distribution of MK2 OE and MK2 KD cells in different phases of the cell cycle was determined by flow cytometry. **(D)** Evaluation of ABC transporter activity in ARP1 and OCI-MY5 WT and OE cells by the means of flow cytometry. **(E)** The cellular apoptosis of ARP1 and OCI-MY5 MK2-WT and OE cells treated with or without bortezomib or doxorubicin. ^*^p < 0.05, ^**^p < 0.01. Data are expressed as mean ± SD.

To further characterize the carcinogenic role of MK2 in MM, lentiviral shRNA transfection technology was utilized to knock down the endogenous expression of the MK2 gene in ARP1 and OCI-MY5 cells. The results of the western blot showed the remarkable impairment of MK2 expression levels in MK2-shRNA transfected MM cells (KD) relative to the controls (Ctrl) (*p* < 0.05) (Figure 2A). As demonstrated in Figure 2B, a significant drop in cell growth rate of OCI-MY5 cells was provoked by silencing MK2 (*p* < 0.05), along with the slight reduction in ARP1 cells. Next, we transfected MM with CRISPR lentiviral activation particles to significantly upregulate the expression of MK2 (*p* < 0.05) (Figure 2A). Cell proliferation rate of MK2-OE cells was obviously increased relative to the control cells in both ARP1 and OCI-MY5 cells (*p* < 0.05) (Figure 2B). These findings proposed that MK2 facilitated MM cell proliferation. Flow cytometry analysis for cell cycle distribution found that the proportions of cells in the G2-M phase were increased after MK2 overexpression (ARP1 WT and ARP1 OE: 8.12 vs. 22.9%; OCI-MY5 WT and OCI-MY5 OE: 2.46 vs. 33.3%) (Figure 2C). Conversely, knockdown of MK2 decreased the proportions of cells in the G2-M phase (ARP1 WT and ARP1 KD: 26.6 vs. 6.51%; OCI-MY5 WT and OCI-MY5 KD: 22.0 vs. 4.13%) (Figure 2C). Furthermore, MK2-OE cells generated significant reduction of the mean fluorescence intensity, revealing a marked increment of ABC transporter substrates efflux (Figure 2D). Meanwhile, the data illustrated that treatment of cells with bortezomib (8 nM) or doxorubicin (100 nM) triggered less apoptotic death in the MK2-OE cells compared with its corresponding control cells in the assay (Figure 2E). These results support the point that MK2 is a powerful factor in promoting MM progression and drug resistance, which could be applied as a novel therapeutic target for cancer treatment.

### MK2 Inhibitor Executes Potential Suppressive Function in MM

**Figure 3:**
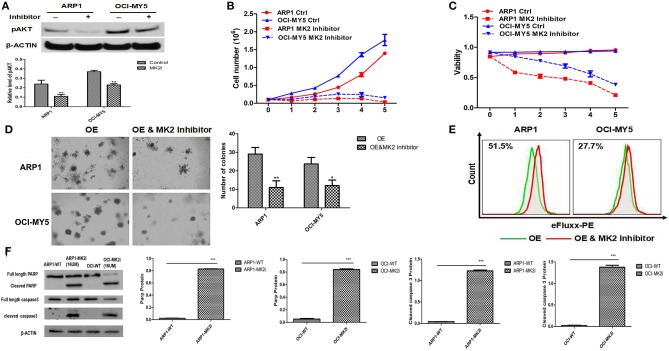
MK2 inhibitor plays a potential suppressive role in MM. **(A)** pAKT expression in ARP1 and OCI-MY5 cells were detected by western blot after MK2 inhibitor IV (15 μM) treatment for 24 h. **(B)** Growth curve of ARP1 and OCI-MY5 cells treated with or without MK2 inhibitor IV for 5 days. **(C)** Cell viability of ARP1 and OCI-MY5 cells treated with or without MK2 inhibitor IV for 5 days. **(D)** The effect of MK2 inhibitor IV treatment on the MK2-OE MM cells compared with control cells was measured by colony formation assay. **(E)** The detection of ABC transport pump activity when treated with MK2 inhibitor IV. **(F)** Western blot of ARP1 and OCI-MY5 cells following MK2 inhibitor IV treatment on the PARP and Caspase-3 expression. Data are expressed as mean ± SD.

To extend our findings into pre-clinics study, we evaluated the effects of MK2 inhibitor IV on MM growth and drug-resistance. MK2 promoted MM progression via activating AKT in our previous study (17). Here we first examined the effect of the MK2 inhibitor on reducing AKT activation. A sharp abolishment on pAKT (the phosphorylation-activated form of AKT) expression was attained by MK2 inhibitor IV treatment, when compared with non-treated control in both ARP1 and OCI-MY5 cells (*p* < 0.05) (Figure 3A), partly suggesting that MK2 plays a positive role in AKT phosphorylation. Subsequently, the growth rate and cell viability of ARP1 and OCI-MY5 cells were significantly hindered by MK2 inhibitor IV compared with their non-treated counterparts in a time-dependent manner (Figures 3B,C). Alternatively, the inhibitor also diminished long-term self-renewal of MK2-OE cells, which was evidenced by the remarkable declined clonogenicity (*p* < 0.05) (Figure 3D). Similarly, ABC transporter drug pump in MK2-OE cells was deactivated by MK2 inhibitor IV treatment, indicating the reverse on MK2 induced drug-resistance (Figure 3E). Combined with the western blot data that MK2 inhibitor IV induced remarkable elevation of cleaved both Caspase-3 and PARP (*p* < 0.05) (Figure 3F), we could speculate that the decreased growth rate of MK2 inhibitor IV treated cells was attributed to the increased apoptotic cell death. Summarily, the data above indicates that MK2 inhibitor IV may act as a potent pre-clinic candidate for targeting MK2 in MM.

### MK2 Inhibitor Synergistically Enhances the Repressive Effect of Bortezomib/Dexamethasone/Doxorubicin on MM Cell Proliferation

**Figure 4:**
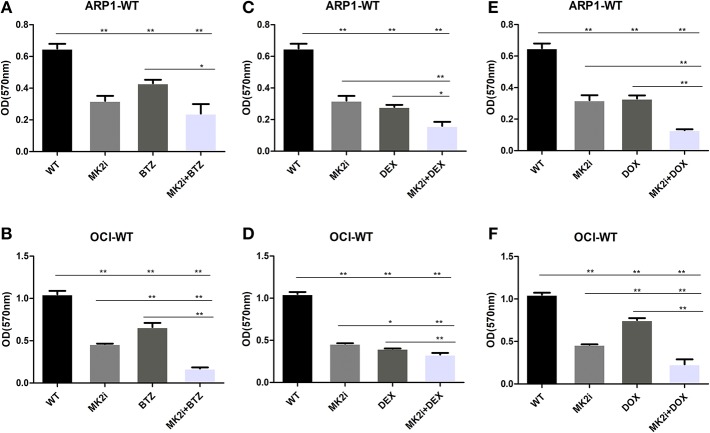
MK2 inhibitor IV enhances the inhibitory effect of bortezomib, dexamethasone, or doxorubicin on cellar proliferation in ARP1 and OCI-MY5. **(A,B)** ARP1 cells **(A)** and OCI-MY5 cells **(B)** were treated with MK2 inhibitor IV with/without BTZ or BTZ alone. **(C,D)** ARP1 cells **(C)** and OCI-MY5cells **(D)** were treated with MK2 inhibitor IV with/without DEX or DEX alone. **(E,F)** ARP1 cells **(E)** and OCI-MY5 cells **(F)** were treated with MK2 inhibitor IV with/without DOX or DOX alone. All the drug cytostatic effect was analyzed by MTT proliferation assay after 48 h. Drug concentrations: MK2 inhibitor IV: 15 μM, bortezomib: 80 nM, dexamethasone: 25 μM, and doxorubicin: 1.5 μM, MK2 inhibitor IV 4.75 μM + bortezomib 46.2 nM, MK2 inhibitor IV 4.75 μM + dexamethasone 6.6 μM, MK2 inhibitor IV 4.75 μM + doxorubicin 0.3 μM. ^*^p < 0.05, ^**^p < 0.01. Data are expressed as mean ± SD.

Bortezomib (BTZ), dexamethasone (DEX), and doxorubicin (DOX) have been well-recognized as key therapeutic agents for MM treatment; hence, MTT assays were performed to examine the effects of the drugs, alone or combined with MK2 inhibitor IV. As shown in Figure 4, when compared with control cells MK2 inhibitor IV and each agent (dexamethasone/doxorubicin/bortezomib) all yielded significant reductions in the growth rates of ARP1 and OCI-MY5 cells (*p* < 0.05). The cell growth inhibitory function of MK2 inhibitor IV was significantly and slightly superior to doxorubicin and bortezomib in OCI-MY5 cells and ARP1 cells, respectively. However, dexamethasone-exposed cells exhibited lower proliferation rate than MK2 inhibitor IV-exposed cells to some extent. Then we explored combined effects of MK2 inhibitor IV and dexamethasone/doxorubicin/bortezomib and found the combinations remarkably suppressed cell proliferation in comparison with the single drug in ARP1 and OCI-MY5 cells (*p* < 0.05). Above all, the data unveiled that MK2 inhibitor is capable of improving bortezomib, dexamethasone, or doxorubicin activity *in vitro*.

### Downregulation of MK2 Improves the Survival Time of 5TMM Mice

**Figure 5:**
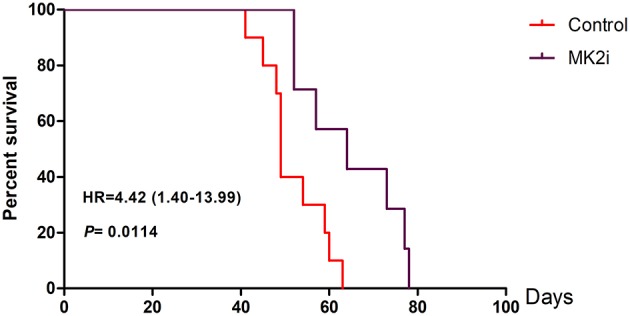
MK2 inhibitor IV exerts a great function on improving survival time of 5TMM mice. MK2 inhibitor IV was intraperitoneally injected at the dose of 20 mg/kg three times a week lasting for 78 days until all the mice were dead (Tuesday, Thursday, and Saturday). Data are expressed as mean ± SD.

To further evaluate the therapeutic potential of the MK2 inhibitor in animal models, survival analyses were performed on 5TMM mice based on the MK2 inhibitor IV (20 mg/kg) treatment. As shown in Figure 5, Kaplan-Meier survival curves showed that mice with high MK2 expression had inferior outcomes than those with low MK2 expression though using MK2 inhibitor IV (*p* < 0.05). Taken together, it further indicates that MK2 may serve as a prognostic marker in MM.

## Discussion

Increasing evidence has shown that activation of p38 MAPK signaling displays a critical role in promoting hematopoietic growth leading to hematologic malignancies in some hematologic systems (18). It has been reported that p38 MAPK regulates Hsp27 through MK2, which confers resistance to bortezomib and dexamethasone in MM cells (19). Amplification of MK2 is closely associated with tumorigenesis and the development of various cancers, like prostate cancer (18), gastrointestinal stromal tumors (20) and bladder cancer (5). However, the role of MK2 in MM is still poorly understood. In the current research, GEP database revealed the impressive increment of MK2 expression levels in MM cells, which is in accordance with the previous study on lung cancer (21). More importantly, patients with high MK2 expression suffered from worse survival outcomes than MK2 low-expressing patients. These studies suggest that MK2 is a novel therapeutic option for MM treatment.

Our work indicated that decreased MK2 expression induced cell growth inhibition, while increased MK2 expression promoted cell proliferation and ABC transporter drug pump activation, which implied a positive function in tumorigenesis and development (Figures 2, 3). It is served as a different angle about the function of MK2 exerted on tumor cells, for the majority reports focus on cell invasion via modulation of matrix metalloproteinase and actin cytoskeleton (5, 7, 22). Inspiringly, inferred from the data associated with MK2 inhibitor IV, we found the inhibitor manifesting remarkable reduction of MM cellular growth, colony formation and ABC transporter drug pump (Figure 3). Furthermore, this process could be mediated by pAKT level (Figure 3A), which was confirmed by our team and other groups (15, 23, 24). However, whether MK2 directly or indirectly phosphorylated AKT in MM cells needs further investigation. In addition, blockade of MK2 activates cleaved PARP and Caspase-3 resulting in triggering cell apoptosis, thus MK2 inhibitor may be a promising agent for MM treatment in clinics.

It has been known that MK2 inhibitors are in preclinical development (25), in which both MK2 inhibitor III and PF-3644022 declined cell survival when combined with mitomycin-c treatment, unlike their pro-survival effects upon application with gemcitabine collectively (26). Meanwhile, cisplatin as well as the topoisomerase II inhibitor doxorubicin combined with MK2 inhibitors were reported as exhibiting increased cytotoxicity (11, 27). In this paper, we traced the influence of MK2 inhibitor IV on increasing the efficacy of bortezomib, doxorubicin, and dexamethasone on MM cell proliferation. The results showed that using MK2 inhibitor IV alone was more effective than bortezomib or doxorubicin. Our study first claimed that combining MK2 inhibitor IV with the anticancer agents (dexamethasone/doxorubicin/bortezomib) had better curative efficacy than single agents. Interestingly, researchers have corroborated that some complex factors like tumor genotype and DNA repair machineries are considered as the manipulators in determining the effect of combination therapy regimens (28, 29). In view of the difficulties in accurate predictions, any cytotoxic drug should be carefully monitored about its collaboration or antagonism with MK2 inhibitors, before applying such drug combinations to the clinics.

In conclusion, our findings illustrate the oncogenetic functions of MK2 in MM, suggesting it is a novel molecular marker. Combining the MK2 inhibitor with bortezomib, doxorubicin or dexamethasone has significant inhibition effect on MM cells. Targeting MK2 may afford a new therapeutic approach to MM. Future studies is undergoing to get better understanding in the interaction of MK2 inhibitor and clinical drugs *in vivo* and elaborate its mechanism at the subcellular level.

## Data Availability

Publicly available datasets were analyzed in this study. This data can be found here: https://www.ncbi.nlm.nih.gov/geo/query/acc.cgi?acc=GSE2658.

## Ethics Statement

This study was carried out in accordance with the recommendations of National Research Council guidance's for the care and use of laboratory animals, and approved by Animal Ethical and Experimental Committee of Nanjing University of Chinese Medicine (ACU170501). The protocol was approved by Animal Ethical and Experimental Committee of Nanjing University of Chinese Medicine.

## Author Contributions

CG and MG conceived the manuscript and provided critical input. MG wrote the manuscript. YYu, YuZ, RW, JH, MS, and YaZ performed the experiments. DS and ZF provided technical counseling on experiments. CG, YYa, JQ, and FL reviewed the data and edited the manuscript. All authors read and approved the final manuscript.

### Conflict of Interest Statement

The authors declare that the research was conducted in the absence of any commercial or financial relationships that could be construed as a potential conflict of interest.

## Abbreviations

MAPK: mitogen-activated protein kinase
MM: multiple myeloma
GEP: gene expression profiling
aCGH: array-based comparative genomichybridization.
